# Lycopene Maintains Mitochondrial Homeostasis to Counteract the Enterotoxicity of Deoxynivalenol

**DOI:** 10.3390/antiox12111958

**Published:** 2023-11-02

**Authors:** Zihui Cai, Fengjuan Chen, Youshuang Wang, Xuebing Wang, Xu Yang, Cong Zhang

**Affiliations:** 1 College of Veterinary Medicine, Henan Agricultural University, No.15 Longzihu University Park, Zhengdong New District, Zhengzhou 450046, Chinafengjuanchen@stu.henau.edu.cn (F.C.); yswang@stu.henau.edu.cn (Y.W.); xbwang74@163.com (X.W.); yangxu@henau.edu.cn (X.Y.); 2 Key Laboratory of Quality and Safety Control of Poultry Products, Ministry of Agriculture and Rural Affairs, Zhengzhou 450046, China

**Keywords:** Deoxynivalenol, mitochondrial homeostasis, Lycopene, IPEC-J2 cells

## Abstract

The intestinal tract is a target organ for Deoxynivalenol (DON) absorption and toxicity. Mitochondrial homeostasis imbalance is the gut toxicity mechanism of DON. Lycopene (LYC) has intestinal protective effects and can maintain mitochondrial homeostasis in response to various danger signals. The purpose of this study was to explore the protective effect of LYC on DON-induced IPEC-J2 cells damage. These results showed that DON exposure induced an increase in the levels of malondialdehyde and reactive oxygen species (ROS) in IPEC-J2 cells. DON impaired IPEC-J2 cell barrier function and caused mitochondrial dysfunction by inducing mitochondrial permeability transition pore (MPTP) opening, mitochondrial membrane potential (MMP) reducing, destroying mitochondrial fission factors, mitochondrial fusion factors, and mitophagy factors expression. However, adding LYC can reduce the toxic effects of DON-induced IPEC-J2 cells and decrease cellular oxidative stress, functional damage, mitochondrial dynamics imbalance, and mitophagy processes. In conclusion, LYC maintains mitochondrial homeostasis to counteract the IPEC-J2 cells’ toxicity of DON.

## 1. Introduction

The food safety threat caused by mycotoxin contamination in natural grains has attracted wide attention. Deoxynivalenol (DON), produced by *Fusarium*, also known as “vomitxin” [1], is widely present in crops, and as one of the most common and harmful mycotoxins in agriculture, has seriously affected human and animal food security [2]. It has been found that 60–70% of wheat is contaminated by DON in the world [3]. DON can accumulate in animal-derived foods via the food chain enrichment effect, seriously endangering human and animal health [2]. DON has toxic effects on the liver, brain, and central nervous system, which can cause diarrhea, anorexia, vomiting, growth retardation, and immunotoxicity [4]. Due to the strong toxic effect of DON, the wide range of contamination has drawn great attention [5].

The intestinal tract, as the first barrier against mycotoxins, is also the main toxic target of DON [6]. The research focused on the relationship between DON and intestinal health has been fruitful in recent years. Studies have found that DON can damage intestinal mucosal epithelium, reduce intestinal villi height, villi height/crypt depth ratio, number of cup cells and lymphocytes, and further disrupt intestinal microbiota homeostasis [7]. The DON-contaminated feed can cause damage to the intestinal barrier of piglets and ultimately lead to growth inhibition [8]. In addition, DON also has extremely strong cytotoxicity, which can inhibit the formation of proteins and ribosomal nucleotides, induce oxidative stress in the endoplasmic reticulum, thus induce cell apoptosis, and ultimately destroy the homeostasis of the intestinal epithelium [9]. There are reports that DON can not only lead to inflammation and apoptosis of intestinal epithelial cells but also destroy the integrity of the intestinal barrier and cause intestinal damage [10]. These results indicated that DON has severe intestinal toxicity, and it is urgent to find out the key targets of the toxic mechanism of DON for the development of therapeutic drugs.

Mitochondria are one of the critical organelles that produce energy and coordinate the metabolism in cells [11]. Mitochondria act as a crucial part of the realization of various intestinal functions and the protective effect of the intestinal barrier. The tight connections formation is highly energy-dependent in the gut, so mitochondria are crucial for facilitating the formation of tight connections [12]. The microvilli structure derived by intestinal epithelial cell differentiation is very important for nutrient absorption, which is maintained by mitochondrial biogenesis. Abnormal mitochondrial biogenesis will affect the differentiation of intestinal epithelial cells, break intestinal homeostasis, and even induce cell death [13]. Mitochondrial oxidative stress has been identified as a key driver of intestinal diseases [14]. In addition, Studies have found that Aloe vera gel can protect the intestinal barrier by reducing the excessive accumulation of reactive oxygen species (ROS) controlled by mitochondria and maintaining mitochondrial function [15]. Mitochondrial quality control plays a key and important role in preserving intestinal health. Impaired mitochondrial quality control can lead to disrupted intestinal homeostasis and further damage to the intestinal barrier permeability [16]. Mitochondrial homeostasis imbalance has been viewed as the key intestinal dysfunction mechanism induced via DON. Therefore, further exploring substances that can maintain mitochondrial homeostasis may become an effective method and potential therapeutic drug for antagonizing the enterotoxicity DON.

Lycopene (LYC) is a carotenoid that is widely found in tomatoes, papaya, watermelon, pomegranate, pink grapefruit, and other red foods and fruits [17]. Due to the polyunsaturated bond structure of LYC, LYC can alleviate intestinal barrier disorders and maintain intestinal health via its antioxidant and anti-inflammatory properties [18]. LYC can be used as an adjunctive medication in the treatment of colorectal cancer and colon cancer [19]. Current studies found that LYC can reduce mitochondrial damage by maintaining mitochondrial redox balance homeostasis, protecting mitochondrial morphological structure, and regulating mitophagy and mitochondrial quality control system, thus achieving the therapeutic effect and a promising effect on protecting cell function [20]. However, whether LYC can decrease mitochondrial damage and maintain mitochondrial homeostasis to mitigate intestinal toxicity caused by DON is unknown.

In this study, the protective effect of LYC on the mitochondrial dysfunction induced using DON was further investigated, and the protective mechanism of LYC on IPEC-J2 cells was elucidated, which offered a new direction for the treatment of mycotoxins and the in-depth study of mitochondrial homeostasis.

## 2. Materials and Methods

### 2.1. Cell Culture and Treatment

DMEM, 15% FBS, 1% penicillin-streptomycin, and 1% glutamine were mixed to supply the culture environment to IPEC-J2 cells. The IPEC-J2 cell, originally isolated from the mid-jejunum of a neonatal unsuckled piglet, was purchased from Tongpai Biotechnology Co., Ltd. (Shanghai, China). DON was purchased from Pribolab (MSS1011; Qingdao, China), and LYC was purchased from Weikeqi Biological Technology Co., Ltd. (502-65-8; Chengdu, China). DON and LYC were first dissolved in dimethyl sulfoxide (DMSO) and then further diluted in the cell culture medium. The DMSO amount in the culture medium did not exceed 0.1% (*v*/*v*), a concentration that did not affect the assays (results were similar to the application of a vehicle-free control medium). Vehicle-free control medium was used as control. According to the experiment requirement, the cells were incubated in cell culture dishes and well plates. The cells were inoculated into different sizes of culture vessels and then cultured in a humidified incubator (37 °C, 5% CO_2_).

### 2.2. Cell Viability Assay

Using CCK-8 to detect cell viability and the detailed operation steps were following kit instructions. For the DON experiment alone, the cells were inoculated into a 96-well plate with an initial density of 2 × 10^4^ cells per well. Then, the cells were treated with LYC at concentrations of 0, 10, 20, 30, 40, 50, 60, 70, 80, 90, and 100 µg/mL. The phosphate-buffered saline (PBS) was filled the hole at the edge to prevent the cell medium from evaporating. After incubating at 37 °C for 24 h, added CCK-8 to each well for 1 h, and then the Infinite M200 FA plate reader (TECAN, Männedorf, Switzerland) was used to measure cell viability.

For the DON + LYC experiment, the cells with an initial density of 2 × 10^4^ cells per well were inoculated into a 96-well plate, and then LYC with concentrations of 0, 10, 20, 30, 40, 50, 60, 70, 80, 90, 100 µg/mL was added together with DON to the cells which concentration was based on a previous study [21]. After 24 h of cell culture, CCK-8 was added to each wall for 1 h, and then the Infinite M200 FA plate reader was used to measure cell viability.

### 2.3. Malondialdehyde (MDA) Assay

The content of MDA in cells was determined according to the Malondialdehyde Microplate Assay Kit (Solarbio Institute of Biotechnology, Beijing, China). Simply, an initial density of 2 × 10^5^ cells per well was inoculated into a 6-well plate. Once they reached about 80 percent confluence, the cells were divided into four groups: CON group (vehicle-free control medium), DON group (148.16 ng /mL DON), LYC + DON group (30 µg/mL LYC + 148.16 ng/mL DON), LYC group (30 µg/mL LYC). After 24 h of inoculation, the cells were centrifuged, and MDA extract solution was added with MDA to obtain MDA from the cells. Then, the cells were centrifuged again and the supernatant was divided into two parts. Some to detect the standard protein concentration of the sample, and the other part was used to detect the MDA content using an MDA detection kit. The standard protein concentration was measured using the BCA. In addition, it can be seen from the kit explanation method that under high temperature and acidic conditions, MDA and thiobarbituric acid (TBA) will produce a red product which has a strong absorption peak at A532 nm. Then, the absorption value was measured at A532 nm using the Infinite M200 FA plate reader.

### 2.4. Reactive Oxygen Species (ROS) Assay

Using Reactive Oxygen Species Assay Kit (Beyotime Institute of Biotechnology, Shanghai, China) to measure the content of ROS in cells. In brief, an initial density of 2 × 10^5^ cells per well was inoculated into a 6-well plate. Once they reached about 80 percent confluence, the cells were divided into four groups for incubation 24 h. Then, the medium was sucked out with a pipette gun. After rinsing with PBS, the fluorescence probe DCFH-DA has been added into the cells, and the cells were incubated in 37 °C with dark environment for 1 h. Finally, fluorescence was measured using EVOS M5000 cell imaging system (AMF5000, ThermoFisher, Shanghai, China).

### 2.5. Mitochondrial Membrane Potential (MMP) Assay

Using a mitochondrial membrane potential assay kit with JC-1 (Beyotime Institute of Biotechnology, Shanghai, China) to measure MMP. Briefly, an initial density of 2 × 10^5^ cells per well was inoculated into a 6-well plate. Once they reached about 80 percent confluence, the cells were divided into four groups for incubation. After 24 h, Then, the medium was sucked out with a pipette gun and the cells were rinsed with PBS, then added the fluorescent probe JC-1 into the primary cells and incubated in 37 °C with dark environment for 20 min. Then, the labeled cells were observed with inverted fluorescence microscope (n = 3).

### 2.6. Mitochondrial Permeability Transition Pore (MPTP) Assay

Using MPTP Assay Kit to measure the open state of MPTP. In brief, an initial density of 2 × 10^5^ cells per well was inoculated into a 6-well plate. Once they reached about 80 percent confluence, the cells were divided into four groups for incubation. After 24 h, the medium was sucked out with a pipette gun, and the cells were rinsed with PBS. Added dye solution and incubated cells at 37 °C for 30 min away from light. Then, the labeled cells were observed with inverted fluorescence microscope (n = 3).

### 2.7. qRT-PCR Analysis

An initial density of 2 × 10^5^ cells per well was inoculated into a 6-well plate. Once they reached about 80 percent confluence, the cells were divided into four groups for incubation 24 h. Three replicates were conducted for each group. After rinsing with PBS, an RNA extraction solution was added to fully lyse the cells. Subsequently, 380 µL of chloroform was used for phase separation and equivoluminal isopropanol for RNA precipitation. Subsequently, RNA was eluted in 20 µL RNase-free water after being washed thrice in 75% ethanol. HiScript III All-in-one RT SuperMix (R232-01, Nanjing Vazyme Biotechnology Co., Ltd., Nanjing, China) was used to reverse transcribe it into cDNA. Finally, Polymerase chain reaction (PCR) amplification and sequencing were analyzed using *qTower 3G (analytik jena, Jena, Germany). The primer sequence is shown in Table S1 (synthesized by Beijing Qingdao Biotechnology Co., Ltd. Beijing, China). The results of qRT-PCR were calculated using 2^−ΔΔCT^ formula.

### 2.8. Western Blot Analysis

IPEC-J2 cells were seeded at 2 × 10^4^ cells/well in 75 cm^2^ flasks. Three replicates were conducted for each group. At the end of the treatment, total proteins were extracted using RIPA buffer (Beyotime Biotechnology) and centrifuged at 12,000 r/min for 10 min, as previously described. The protein concentration in the supernatant was determined using BCA assay kit (Beyotime Institute of Biotechnology, Shanghai, China). 30 µg protein per sample was separated using 12% SDS-PAGE and transferred to the PVDF membrane (Millipore Corporation, Billerica, MA, USA). Next, the membranes were blocked with non-fat milk at room temperature, and incubated with primary antibodies (Anti-ZO-1 (diluted 1:1750, WL03419, Wanleibio), Anti-Occludin (diluted 1:1250, WL01996, Wanleibio), Anti-claudin-1 (diluted 1:1750, WL03073, Wanleibio), Anti-N-cad (diluted 1:1000, A19083, ABclonal), Anti-Parkin (diluted 1:500, WL02512, Wanleibio), Anti-PINK1 (diluted 1:750, WL04963, Wanleibio), Anti-Dnm1 (diluted 1:1750, WL03028, Wanleibio); β-actin (diluted 1:2000, GB12001, Servicebio) at 4 °C overnight. The secondary antibody (diluted 1:5000, AS014, ABclonal) was then used to incubate the membrane. Finally, the protein bands were imaged using the Amersham Imager 600 system with an ECL reagents kit (Applygen, Beijing, China) and analyzed with Image J software (version 1.8.0).

### 2.9. Data Statistics and Analysis

All experimental results were collected using at least three independent experiments. The experimental data were analyzed using GraphPad Prism (version 5.0) and SPSS (version 22.0). The results were calculated with mean ± SEM. All data were analyzed using one-way analysis of variance (ANOVA); *p* < 0.05 was considered to be statistically significant.

## 3. Results

### 3.1. LYC Alleviated DON Induced IPEC-J2 Cells Damage

**Figure 1:**
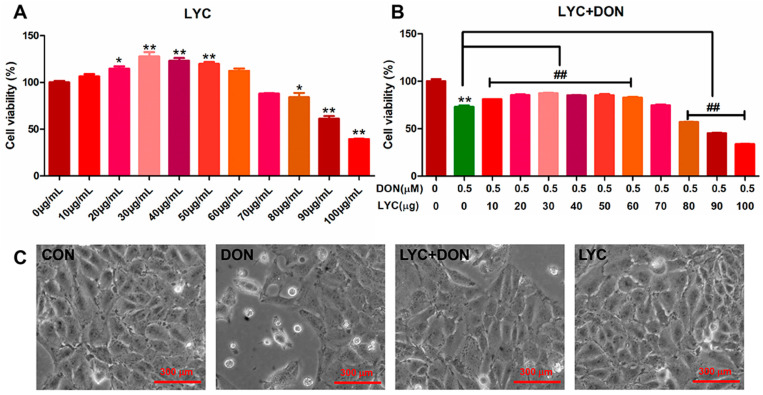
LYC alleviated the cytotoxicity of IPEC-J2 induced using DON. (**A**) Changes in cell activity under different concentrations of LYC (10, 20, 30, 40, 50, 60, 70, 80, 90, and 100 μg/mL). (**B**) Changes in cell activity of LYC (10, 20, 30, 40, 50, 60, 70, 80, 90, and 100 μg/mL) combined with DON (148.16 ng/mL). (**C**) Cell morphology of IPEC-J2 cells cultured with DON and/or LYC for 24 h, respectively. Each reported value (n = 5) represents the mean ± SEM. Asterisk (*) for the significance of differences between the control and another, * *p* < 0.05, ** *p* < 0.01: Hashes (#) for the significance of differences between the DON and LYC + DON, ^##^ *p* < 0.01.

CCK-8 kit was used to detect the effect of LYC and/or DON on the cell viability of IPEC-J2cells. Compared with the control group, with the continuous increase in LYC concentration, cell viability showed a trend of first increasing and then decreasing. When the concentration of LYC was in the range of 10–60 µg/mL, the cell activity was enhanced. 30 µg/mL LYC resulted in the highest cell viability of IPEC-J2 cells. However, when the dose exceeded 80 µg/mL, the cell activity decreased significantly. So, LYC concentration was used as 30 µg/mL in subsequent experiments (see Figure 1A).

Our previous study found that DON exposure could significantly reduce IPEC-J2 cell survival. According to the results above, we chose the DON dose to be 148.16 ng/mL. To verify the protective effect of LYC on DON cell activity, we treated IPEC-J2 cells with different concentrations of LYC (10, 20, 30, 40, 50, 60, 70, 80, 90, and 100 µg/mL) and DON 148.16 ng/mL, respectively. Compared with the control group, the LYC treatment group alleviated cell damage caused by DON, and cell activity was increased to the highest level when the LYC concentration was 30 µg/mL (see Figure 1B). Therefore, the optimal concentration of LYC was selected as 30 µg/mL for subsequent experiments. In addition, via the observation of cell morphology, it could be obviously seen that DON exposure will cause flat and sparse cells, and the intervention of LYC could reduce the cytotoxicity of DON and protect the cell morphology.

### 3.2. LYC Alleviated DON Induced Intestinal Epithelial Barrier Impairment in IPEC-J2 Cells

**Figure 2:**
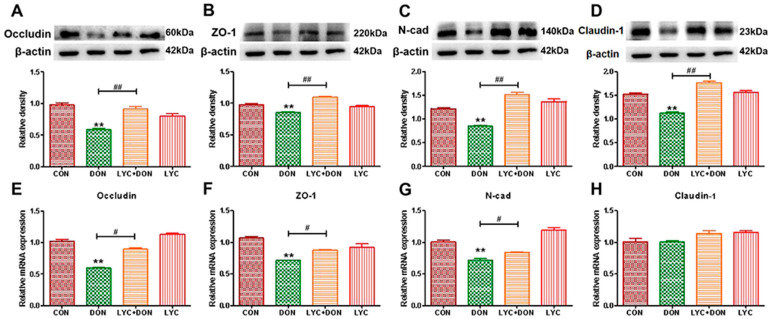
LYC alleviated the intestinal barrier damage induced using DON. (**A**) Western-blot analysis of Occludin. (**B**) Western blot analysis of ZO-1 protein. (**C**) Western-blot analysis of N-cad protein. (**D**) Western-blot analysis of Claudin-1 protein. (**E**) Relative RNA abundance of Occludin. (**F**) Relative RNA abundance of ZO-1. (**G**) The relative RNA abundance of N-cad. (**H**) Relative RNA abundance of Claudin-1. IPEC-J2 cells were divided into CON, DON, LYC + DON, and LYC groups. Each reported value (n = 3) represents the mean ± SEM. Asterisk (*) for the significance of differences between the control and another, ** *p* < 0.01: Hashes (#) for the significance of differences between the DON and LYC + DON, ^#^ *p* < 0.05, ^##^ *p* < 0.01.

To demonstrate the protective effect of LYC- on DON-induced intestinal epithelial barrier impairment in IPEC-J2 cells, we selected qRT-PCR and Western-blot analysis to determine the expression of intestinal epithelial barrier-related proteins. Compared with the control group, the gene and protein expression of Occludin, ZO-1, Claudin-1, and N-cad in the DON group decreased (see Figure 2). At the same time, LYC treatment can reverse this trend and restore the expression of intestinal epithelial barrier-related proteins and genes to a relatively normal level. These results deduced that LYC alleviated DON-induced intestinal epithelial barrier impairment in IPEC-J2 cells.

### 3.3. LYC Alleviated DON Induced Oxidative Stress in IPEC-J2 Cells

**Figure 3:**
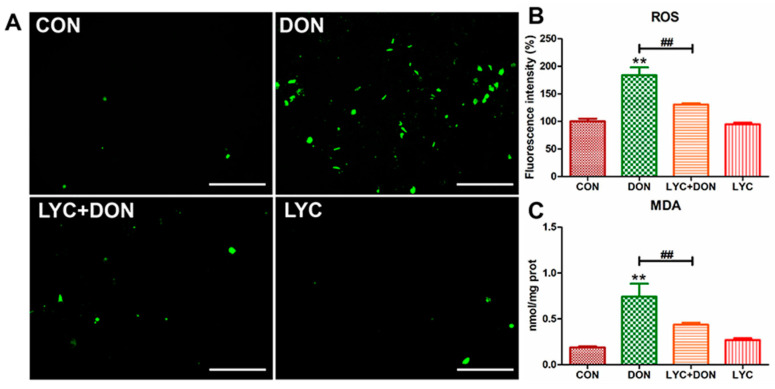
LYC alleviated the oxidative damage of IPEC-J2 cells caused by DON. (**A**) Images of ROS fluorescence. Scale bar = 500 µm (**B**) ROS content. (**C**) MDA content. IPEC-J2 cells were divided into CON, DON, LYC + DON, and LYC groups. Each reported value (n = 3) represents the mean ± SEM. Asterisk (*) for the significance of differences between the control and another, ** *p* < 0.01: Hashes (#) for the significance of differences between the DON and LYC + DON, ^##^ *p* < 0.01.

To analyze the alleviating effect of LYC on the oxidative stress of IPEC-J2 cells induced using DON, we detected the ROS content in IPEC-J2 cells with a DCFH-DA fluorescent probe and detected the MDA content in the cells with an MDA detection kit. The result was shown in Figure 3; the ROS level in the cells of the DON group was clearly increased compared with the control group. At the same time, LYC treatment can reverse this trend and return the ROS level to a relatively normal level (see Figure 3B), which can be intuitively seen from the fluorescence pictures (see Figure 3A). In addition, MDA content in the DON group increased visibly, and the trend returned to a nearly normal level after LYC treatment compared with the control group (see Figure 3C). These results indicated that LYC had a good therapeutic effect on DON-induced oxidative damage in IPEC-J2 cells.

### 3.4. LYC Alleviated DON Induced Mitochondrial Impairment in IPEC-J2 Cells

**Figure 4:**
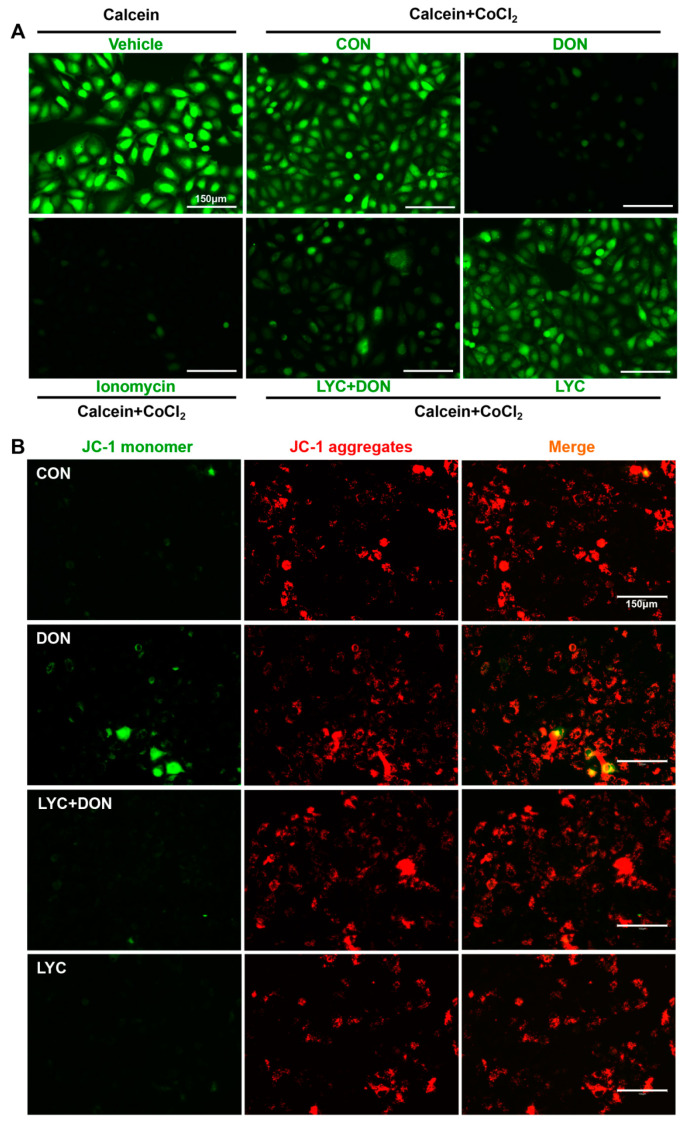
LYC alleviated the DON-induced mitochondrial damage. IPEC-J2 cells were divided into CON, DON, LYC + DON, and LYC groups. (**A**) Mitochondrial membrane potential (MMP) in IPEC-J2 cells. (**B**) Mitochondrial permeability transition pore (mPTP) opening in IPEC-J2 cells. Scale bar = 150 µm.

Calcein AM is a non-polar dye that can fluorescently stain living cells. It can easily penetrate the living cell membrane and be hydrolyzed into Calcein by intracellular esterase, thus remaining in the cell and stimulating strong green fluorescence. In addition, CoCl_2_ can quench the green fluorescence of Calcein in the cytoplasm. When MPTP is turned off, CoCl_2_ cannot enter the mitochondria. Therefore, Calcein AM staining will cause the green fluorescence of Calcein. On the contrary, when MPTP is open, CoCl_2_ will enter the mitochondria and partially or even completely quench the fluorescence of Calcein. Eventually, the green fluorescence weakens or even disappears. As shown in Figure 4A, the MPTP opening was activated, and CoCl_2_ entered the mitochondria, thereby quenching the green fluorescence of Calcein in the DON group. However, this phenomenon was alleviated after LYC treatment. The above results indicated that LYC could reverse DON-induced abnormal MPTP opening in IPEC-J2 cells.

JC-1 is a permeable membrane cationic fluorescent dye, which is often used in the detection of MMP. JC-1 exists in two forms: monomer and polymer. When MMP is normal, JC-1 exists in the form of a polymer, showing red fluorescence. However, when MMP is decreased, JC-1 will be released from the mitochondria. The concentration of JC-1 decreases, and the fluorescence changes from red to green. As shown in Figure 4B, DON exposure reduced the red fluorescence and enhanced the green fluorescence. This indicated that DON has a damaging effect on MMP. However, LYC treatment could reverse this phenomenon and alleviate the MMP decrease induced using DON.

### 3.5. LYC Alleviated DON Induced Mitochondrial Dynamics Disturbance in IPEC-J2 Cells

**Figure 5:**
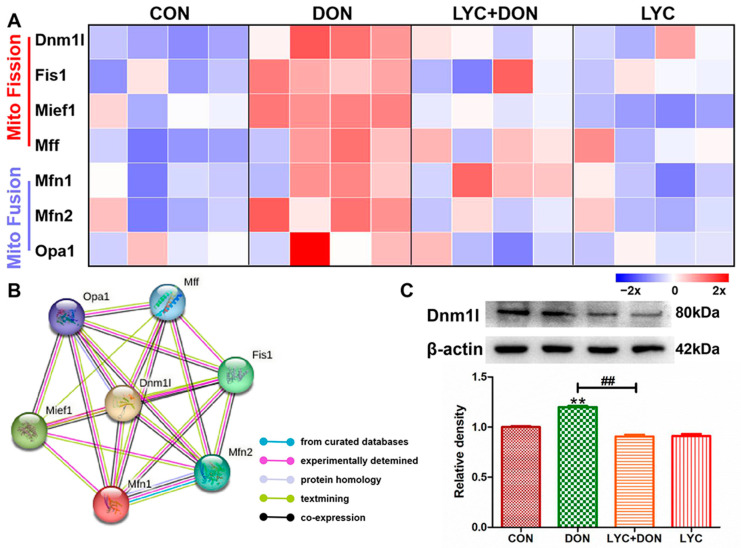
LYC alleviated the mitochondrial dynamics disturbance induced using DON. IPEC-J2 cells were divided into CON, DON, LYC + DON, and LYC groups. (**A**) The relative RNA abundance of mitochondrial fission-related genes (Dnm1, Fis1, Mief1, Mff) and the relative RNA abundance of mitochondrial fusion-related genes (Mfn1, Mfn2, OPA1). (**B**) The PPI network of mitochondrial fission and fusion-related genes was constructed using the STRING 10 database. (**C**) Western blot analysis of Dnm1 protein. Each reported value (n = 3) represents the mean ± SEM. Asterisk (*) for the significance of differences between the control and another, ** *p* < 0.01: Hashes (#) for the significance of differences between the DON and LYC + DON, ^##^ *p* < 0.01.

To explore the alleviating effect of LYC on the disturbance of mitochondrial dynamics in IPEC-J2 cells induced using DON, we used Western blot and qRT-PCR to detect the expression of proteins and genes related to mitochondrial dynamics. As shown in Figure 5A, compared with the control group, the expression levels of mitochondrial fusion-related genes Opa1, Mfn1, and Mfn2 were significantly decreased; meanwhile, mitochondrial fission-related genes Mff, Mief1, Fis1, and Dnm1l were significantly increased in DON group compared with the control group. However, LYC treatment could well reverse this trend to maintain the balance between mitochondrial fission and fusion. The PPI network of mitochondrial fission and fusion-related genes was constructed using the STRING 10 database. As shown in Figure 5B, Dnm1l is the core protein in the network. Furthermore, LYC can also reduce DON-induced overexpression of Dnm1l protein compared with a control group (see Figure 5C). These results deduced that LYC can effectively alleviate DON-induced mitochondrial dynamics disorder in IPEC-J2 cells.

### 3.6. LYC Alleviated DON Induced Mitophagy in IPEC-J2 Cell

**Figure 6:**
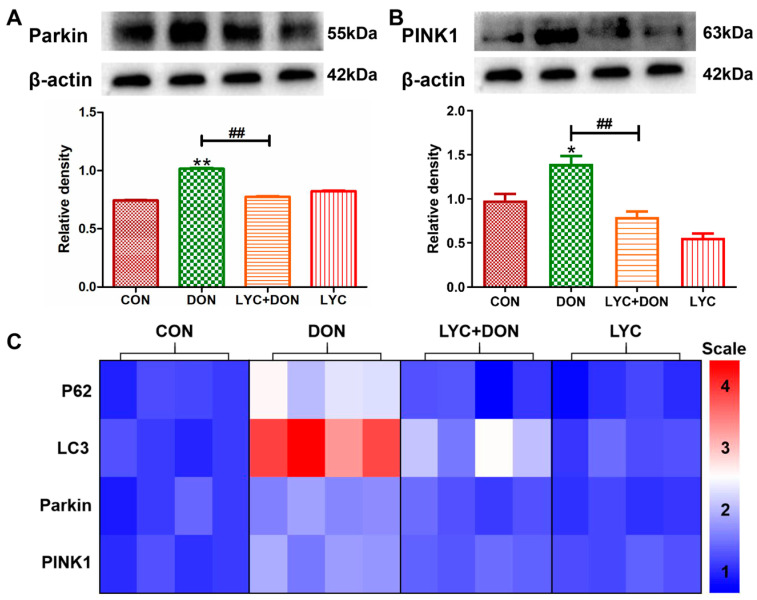
LYC alleviated the mitophagy imbalance induced using DON. IPEC-J2 cells were divided into CON, DON, LYC + DON, and LYC groups. (**A**) Western blot analysis of Parkin protein. (**B**) Western blot analysis of PINK1 protein. (**C**) Relative RNA abundance of mitophagy-associated factors (P62, LC3, Parkin, PINK1). Each reported value (n = 3) represents the mean ± SEM. Asterisk (*) for the significance of differences between the control and another, * *p* < 0.05, ** *p* < 0.01: Hashes (#) for the significance of differences between the DON and LYC + DON, ^##^ *p* < 0.01.

To verify the protective effect of LYC on DON-induced IPEC-J2 mitophagy, we examined mRNA and protein levels for markers of mitophagy. Compared with the control group, the protein expressions of Parkin and PINK1 were increased clearly in the DON group. This suggested that DON exposure can induce abnormal mitophagy; however, LYC addition therapy can reverse this trend and restore the expression of autophagy markers to relatively normal levels (see Figure 6A,B). At the same time, in the DON group, the expressions of Parkin, PINK1, LC3, and P62 genes were also significantly increased. However, after LYC treatment, these changes were significantly alleviated (see Figure 6C). In conclusion, these data indicated that LYC can mitigate the activation of IPEC-J2 cell mitosis after DON exposure and thus maintain mitophagy balance.

### 3.7. Correlation and PCA Analysis

**Figure 7:**
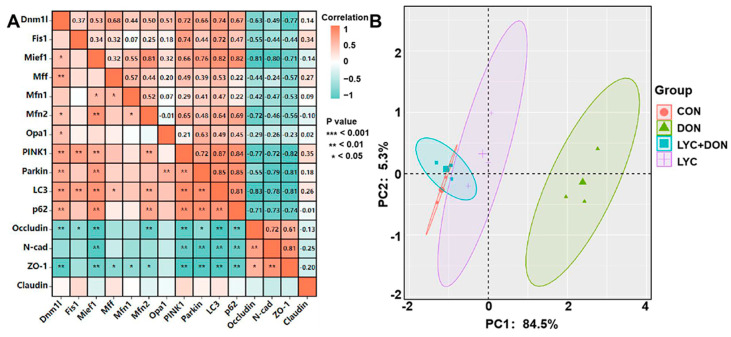
Correlation and PCA analysis. (**A**): Correlation analysis between mitochondrial dynamics protein, mitophagy, and TJ related protein; (**B**): PCA score plot results comparing the mitochondrial dynamics, mitophagy, and TJ related genes expression in IPEC-J2 cells.

To further illustrate the protective mechanism of LYC on DON-induced IPEC-J2 cell damage, we conducted correlation and PCA analysis on mitochondrial dynamics, mitophagy, and tight junction (TJ) related gene expression (see Figure 7). Pearson’s correlation coefficients showed that there were strongly positive correlations between the mitochondrial dynamics-related factors (Dnm1l and Mief1) and mitophagy-related genes (*p* < 0.01). On the contrary, mitochondrial dynamics-related factors (Dnm1l and Mief1) and mitophagy-related genes (Parkin, PINK1, LC3, and P62) had a strongly negative correlation with TJ related genes (Occludin, N-cad, and ZO-1) (*p* < 0.05) (see Figure 7A). In addition, PCA score plot results showed the distance between the CON group and the LYC + DON group. Moreover, there was an overlap among the CON, LYC, and LYC + DON groups (see Figure 7B). Together, these data indicated that LYC alleviated DON-induced IPEC-J2 cell damage via regulating mitochondrial dynamics and mitophagy-related gene expressions.

## 4. Discussion

The gut is the main absorption and target of DON [22]. It has been proven that mitochondria are the key target of DON intestinal toxicity. Therefore, it is particularly important to explore drugs that can alleviate the mitochondrial damage induced using DON. LYC is a fat-soluble carotenoid with a strong mitochondrial protective function. LYC has a protective effect on mitochondrial and intestinal health [23]. In this study, the protective mechanism of LYC against mitochondrial damage induced using DON in IPEC-J2 cells was revealed, and it will offer a new direction for applying drug therapy of LYC on DON enterotoxicity.

Porcine is sensitive to DON exposure. Hence, the IPEC-J2 cells were used as research subjects in this study. We treated IPEC-J2 cells with different concentrations of LYC and cultured them for 24 h to screen suitable concentrations and found that the cell activity was the highest at 30 μg/mL. Meanwhile, the different concentrations of LYC (10–60 μg/mL) can effectively alleviate the cytotoxicity by DON (148.16 ng/mL). Hence, 30 μg/mL LYC and 148.16 ng/mL DON were used to uncover the mechanism of LYC alleviating the intestinal toxicity of DON. The intestinal barrier is mainly composed of a single layer of epithelial cells, which are closely connected together to maintain the intestinal barrier homeostasis [24]. The intestinal barrier is very vulnerable to damage under various external adverse factors. It was confirmed that DON had a toxic effect on IPEC-J2 cells by morphological observation, and DON can induce the increase in intercellular space and the destruction of intercellular connections. After LYC treatment, the cytotoxicity induced using DON was significantly alleviated, the cell morphology was restored, the cell space was reduced, and the tight intercellular connection was protected. The changes in cell morphology demonstrated that LYC can alleviate cell connection damage caused by DON. TJ protein has a crucial effect on maintaining intestinal health in intestinal epithelial cells, and the stability of the intestinal barrier is closely decided by the stable expression of TJ protein. Occludin and Claudin-1 are the nuclear components of the TJ barrier and are involved in the regulation of ion selectivity and permeability of paracellular pathways between adherent cells. N-cadherin (N-cad) is a major transmembrane protein of adhesion, belonging to the classical cadherin family of Ca^2+^-dependent adhesion proteins, which is responsible for regulating the formation and function of adhesion [25]. ZO-1 is a major scaffold protein of TJ, which can interact with other TJ proteins, such as Occludin and Claudin-1, to form complexes mediating TJ and signal transmission between cells [26]. Therefore, Claudin-1, Occludin, N-cad, and ZO-1 levels can reflect the TJ degree of IPEC-J2 cells. In this study, DON could obviously decrease the expression of TJ protein, which proved that DON can induce intestinal barrier dysfunction [27]. After the addition of LYC, the expression levels of TJ-related protein and gene increased and recovered to nearly normal levels, which indicated that LYC could reverse intestinal TJ damage caused by DON and recover the function of the intestinal barrier. Combined with the results of cell activity, cell morphology, and expression of TJ protein, LYC can effectively alleviate the toxic injury of DON on IPEC-J2 cells.

Mitochondria, as the main producer of ROS, are the toxic effect target of DON [28]. ROS accumulation will result in intestinal inflammatory diseases and eventually lead to abnormalities in the intestinal barrier and immune regulation [29]. DON exposure can induce intestinal oxidative stress damage in mice, reduce the expression of total antioxidant capacity, and eventually lead to intestinal injury [30]. In addition, similar studies have found that DON exposure could induce liver oxidative stress injury [5]. In this study, DON not only promoted the production of ROS but caused lipid peroxidation, suggesting that DON can cause oxidative damage to IPEC-J2 cells. However, LYC can alleviate oxidative damage of IPEC-J2 cells by reducing the abnormal production of ROS and MDA induced using DON. Meanwhile, previous study has confirmed that LYC can enhance the activity of antioxidant enzymes such as glutathione peroxidase (GSH-Px) and glutathione S-transferase (GST) to reduce the production of peroxides such as MDA and hydrogen peroxide (H_2_O_2_) [31]. Therefore, we speculated that LYC can improve the antioxidant capacity to alleviate the oxidative damage of IPEC-J2 cells induced using DON. In brief, the addition of LYC could alleviate DON-induced oxidative damage in IPEC-J2 cells. MMP is an important index and mitochondrial function marker that is involved in mitochondrial oxidative phosphorylation, reflecting the redox state of cells. Stable MMP is the basis of mitochondrial ATP production and is used as a sensitive indicator of mitochondrial integrity [32]. MPTP is a channel of the mitochondrial inner membrane and serves as another indicator of mitochondrial function. When the mitochondria are under stress conditions, MPTP will open [33]. The abnormal opening of MPTP will result in the release of mitochondrial matrix contents and finally initiate the process of cell damage [33]. Our findings confirmed that DON can induce the decrease in MMP, the abnormal opening of MPTP, and mitochondrial dysfunction, further aggravating oxidative stress damage. Researchers have discovered that LYC can reduce the liver toxicity of aflatoxin B1 in broilers by reducing mitochondrial damage, including stimulating mitochondrial antioxidant capacity and maintaining mitochondrial biogenesis [34]. Another study has shown that LYC can reduce lipopolysaccharide (LPS)-stimulated ROS overproduction and MMP loss, thereby alleviating mitochondrial oxidative damage and preventing neuroinflammation-related diseases [35]. Our results confirmed that the addition of LYC can alleviate the mitochondrial dysfunction induced using DON and prevent the decrease in MMP and the abnormal opening of MPTP. Cell ROS overproduction is associated with the destruction of MMP and abnormal opening of MPTP [36]. Based on this evidence, we deduced that LYC alleviated oxidative damage in IPEC-J2 cells by mitigating mitochondrial damage.

Mitochondria are highly dynamic organelles, and mitochondria function not only present by the production of ROS, MMP, and MPTP but also by mitophagy, mitochondrial fission, and fusion stability [37]. In healthy conditions, mitophagy is a self-protective mechanism of cell mitochondrial homeostasis. Parkin/PINK1 signaling pathway, as the main regulatory factor of mitophagy, is closely related to mitochondrial quality control, and Dnm1l is a key regulatory factor of mitochondrial fission [38]. OPA1 is an important factor in the inner mitochondrial membrane responsible for the regulation of mitochondrial fusion. In addition, Mfn1/Mfn2 is a factor related to mitochondrial outer membrane fusion. When the Mfn1/Mfn2 ratio and the mitochondrial fusion/fission are imbalanced, it will induce retinal degeneration using mitophagy [39]. DON can inhibit mitochondrial fusion by inducing excessive ROS generation, and increase the expression of mitophagy marker protein (LC3 and p62) at the gene level, further aggravating mitophagy in spleen lymph nodes of porcine [40]. When DON is exposed to IPEC-J2 cells, it causes the cell to overstress and increase mitochondrial fission, thus aggravating mitochondrial damage and eventually leading to the damage of the IPEC-J2 cell TJ barrier [41]. In this study, DON exposure caused mitochondrial dynamics disorder and increased the expression of fission-critical protein (Dnm1l, Fis1, Mief1, Mff) and mitophagy-related protein (Parkin, PINK1, LC3, and p62) while decreased the expression of fusion-related genes (OPA1, Mfn1, and Mfn2). These results suggested that DON exposure led to disruption of mitochondrial dynamics and initiated mitophagy. At present, there has been a lot of progress in the research on the mitochondrial dynamics and mitophagy of LYC. Studies have shown that LYC protects mitochondrial function by maintaining the balance of mitophagy and mitochondrial dynamics, thus inhibiting DEHP-induced oxidative stress damage in the heart [42]. However, at present, the mechanism of whether LYC can alleviate mitochondrial dynamics and autophagy disorders induced using DON has not been fully elucidated, and the relationship between LYC and mitochondria homeostasis is still unclear. In this study, we found that LYC can protect against the inhibition of mitochondrial fission and fusion induced using DON. DON decreased the expression of mitochondrial fusion-related factors, including Mfn1, Mfn2, and OPA1, while increasing the expression of mitochondrial-fission-related factors, including Dnm1l, Fis1, Mief1, and Mff. The results of LYC treatment confirmed that LYC can protect against the imbalanced expression of mitochondrial fission and fusion. The PPI analysis showed that Dnm1l is a dominant molecule in LYC to alleviate DON-induced mitochondrial dynamics disorder. How LYC regulates Dnm1l to restore mitochondrial homeostasis is worth further attention. At the same time, LYC can also inhibit the expression of mitophagy-related factors (Parkin, PINK1, LC3, and p62). These results proved that LYC can relieve mitochondrial dynamic imbalance caused by DON to maintain mitochondrial homeostasis. The inhibitory effect of LYC on mitophagy may depend on (1) LYC treatment alleviates mitochondrial damage and maintains mitochondrial homeostasis or (2) the over-mitophagy process induced using DON has been controlled. The role of mitophagy in alleviating DON enterotoxicity in LYC still needs further exploration.

## 5. Conclusions

**Figure 8:**
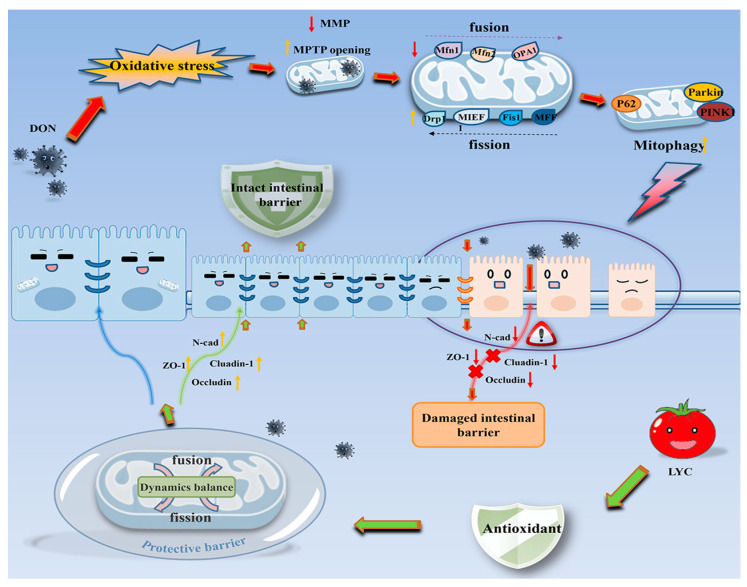
Schematic representation of the proposed mechanism of LYC maintains mitochondrial homeostasis to counteract the enterotoxicity of DON.

DON caused the IPEC-J2 cell damage by inducing mitochondrial dysfunction, which further led to oxidative stress and mitochondrial dynamics imbalance. The intervention of LYC protected mitochondrial homeostasis and function, reduced oxidative stress levels, and alleviated the damage of DON to IPEC-J2 cells (see Figure 8).

In this study, the mechanism of LYC on DON-induced IPEC-J2 cell mitochondrial damage was further investigated. LYC can be used as a feed additive to prevent and control the toxic effect of DON. It is of reference significance for the development of mycotoxin therapy drugs in the future.

## Data Availability

Data are contained within this article and Supplementary Materials.

## Supplementary Materials

The following supporting information can be downloaded at: https://www.mdpi.com/article/10.3390/antiox12111958/s1, Table S1: Sequences of oligonucleotide primers for qRT-PCR.; Table S2: The result of correlation analysis; Figure S1: Original western blot gels used in the study.
