# Supplementary material for: Lycopene Maintains Mitochondrial Homeostasis to Counteract the Enterotoxicity of Deoxynivalenol
Source: Antioxidants (Basel). 2023 Nov 2;12(11):1958. doi: 10.3390/antiox12111958 (PMC10669674; doi:10.3390/antiox12111958)
Supplement: Supplementary file 1 [file antioxidants-12-01958-s001.zip › antioxidants-2658253-supplementary.pdf]

Table S1. Sequences of oligonucleotide primers for qRT-PCR.

Gene Names	Sequence (5' → 3')	NCBI Reference Sequence	Amplicon size (bp)
GAPDH1	GTCGGAGTGAACGGATTTGGC<br>CTTGCCGTGGGTGGAATCAT	NM_001289745.3	150
GAPDH2	AGTATGATTCCACCCACGGC<br>TACGTAGCACCAGCATCACC	NM_001256799.3	139
Occludin	GACAGACTACACAACTGGCGG<br>TGTACTCCTGCAGGCCACTG	NM_001302926.2	662
N-Cad	CTTCATGCTGGTGGTGTCCTTGG<br>CACAGTCTTTGGCAGGGCTCAG	NM_000165.5	149
Claudin	CCATCGTCAGCACCGCACTG<br>CGACACGCAGGACATCCACAG	NM_001244539.1	107
OPA1	ACAGAGGATGGTGCTTGTTGAC<br>CAGTATGATGGCGTTGGGATT	XM_047448216.1	130
Mfn1	AGAAAGCACAAAGCACAGGGGATG<br>CACTGCTGACTGCGAGATACACTC	XM_011512963.4	126
Mfn2	GCCACACCACCAACTGCTTCC<br>TCTTGACGCTCCTCTTCTCCTCTG	XM_047436156.1	96
Dnm1l	TCTGAATCTGGTGGGCATGATTGC<br>CTCCGCAGTAAAGGACTCGAAGTG	XM_001928848.6	91
Fis1	CTACCCAAAGGGAGCAAAGAG<br>GTCCAATGAGTCCAGCCAGTC	NM_016068.3	250
MIEF1	CAGCCGACCACATCCAACT<br>CGAATCACATAGTCCAAGAGGG	NM_001394030.1	271
MFF	GACCTCCTCCAACCCCTCAAAATG<br>GACTAGCTGTCCATTCTGGCGAAC	XM_047445133.1	104
Parkin	GTGGTTCTGCGGTGGATTCTGAG<br>TTACTGCCTGTGGTTCTTTGGGAAG	NM_001044603.2	104
PINK1	CAGGAGCGGTCCCAAGCA<br>GTCATCACAGTGGCGAGGC	NM_032409.3	246
LC3B	TGCCCTCAGACCGGCCTTTCAAGCA<br>TCCTTCTCCTGCTCATAGATGTCCG	XM_047440559.1	310
P62	CGATGGCGATGTCGTATGTG<br>TTGCTGTGCTCCTTGTGAATG	XM_003123639.4	244

Table S2. The result of correlation analysis.

		Dnm1l	Fis1	Mief1	Mff	Mfn1	Mfn2	Opa1	PINK1	Parkin	LC3	P62	Occludin	N-cad	ZO-1	Claudin
Dnm1l	Pearson correlation	1	.372	.528*	.685**	.440	.505*	.510*	.716**	.655**	.740**	.669**	-.635**	-.492	-.771**	.139
	Significant（Bilateral）		.156	.035	.003	.088	.046	.044	.002	.006	.001	.005	.008	.053	.000	.608
	N	16	16	16	16	16	16	16	16	16	16	16	16	16	16	16
Fis1	Pearson correlation	.372	1	.337	.318	.068	.250	.182	.740**	.437	.716**	.465	-.552*	-.438	-.438	.344
	Significant（Bilateral）	.156		.202	.229	.803	.350	.501	.001	.090	.002	.069	.027	.090	.089	.192
	N	16	16	16	16	16	16	16	16	16	16	16	16	16	16	16
Mief1	Pearson correlation	.528*	.337	1	.322	.549*	.813*	.319	.658**	.764**	.815**	.819**	-.811**	-.797**	-.712**	-.143
	Significant（Bilateral）	.035	.202		.224	.028	.000	.228	.006	.001	.000	.000	.000	.000	.002	.598
	N	16	16	16	16	16	16	16	16	16	16	16	16	16	16	16
Mff	Pearson correlation	.685**	.318	.322	1	.567*	.440	.199	.493	.389	.533*	.221	-.438	-.239	-.568*	.274
	Significant（Bilateral）	.003	.229	.224		.022	.088	.461	.053	.136	.034	.410	.090	.373	.022	.304
	N	16	16	16	16	16	16	16	16	16	16	16	16	16	16	16
Mfn1	Pearson correlation	.440	.068	.549*	.567*	1	.520*	.067	.292	.408	.471	.225	-.417	-.471	-.533*	.095
	Significant（Bilateral）	.088	.803	.028	.022		.039	.806	.272	.117	.066	.402	.108	.065	.033	.727
	N	16	16	16	16	16	16	16	16	16	16	16	16	16	16	16
Mfn2	Pearson correlation	.505*	.250	.813**	.440	.520*	1	-.009	.648**	.481	.644**	.694**	-.717**	-.458	-.565*	-.099
	Significant（Bilateral）	.046	.350	.000	.088	.039		.974	.007	.059	.007	.003	.002	.075	.023	.716
	N	16	16	16	16	16	16	16	16	16	16	16	16	16	16	16
Opa1	Pearson correlation	.510*	.182	.319	.199	.067	-.009	1	.207	.628**	.487	.445	-285	-.263	-.233	.015
	Significant（Bilateral）	.044	.501	.228	.461	.806	.974		.442	.009	.056	.084	.284	.326	.384	.955
	N	16	16	16	16	16	16	16	16	16	16	16	16	16	16	16
PINK1	Pearson correlation	.716**	.740**	.658**	.493	.292	.648**	.207	1	.724**	.868**	.839**	-.770**	-.723**	-.820**	.348
	Significant（Bilateral）	.002	.001	.006	.053	.272	.007	.442		.002	.000	.000	.000	.002	.000	.187
	N	16	16	16	16	16	16	16	16	16	16	16	16	16	16	16
Parkin	Pearson correlation	.655**	.437	.764**	.389	.408	.481	.628**	.724**	1	.851**	.846**	-.552*	-.790**	-.814**	.177
	Significant（Bilateral）	.006	.090	.001	.136	.117	.059	.009	.002		.000	.000	.027	.000	.000	.512
	N	16	16	16	16	16	16	16	16	16	16	16	16	16	16	16
LC3	Pearson correlation	.740**	.716**	.815**	.533*	.471	.644**	.487	.868**	.851**	1	.813**	-.829**	-.775**	-.806**	.260
	Significant（Bilateral）	.001	.002	.000	.034	.066	.007	.056	.000	.000		.000	.000	.000	.000	.331
	N	16	16	16	16	16	16	16	16	16	16	16	16	16	16	16
P62	Pearson correlation	.669**	.465	.819**	.221	.225	.694**	.445	.839**	.846**	.813**	1	-.715**	-.728**	-.741**	-.011
	Significant（Bilateral）	.005	.069	.000	.410	.402	.003	.084	.000	.000	.000		.002	.001	.001	.969
	N	16	16	16	16	16	16	16	16	16	16	16	16	16	16	16
Occludin	Pearson correlation	-.635**	-.552*	-.811**	-.438	-.417	-.717**	-.285	-.770**	-.552*	-.829**	-.715**	1	.722**	.610*	-.134
	Significant（Bilateral）	.008	.027	.000	.090	.108	.002	.284	.000	.027	.000	.002		.002	.012	.621
	N	16	16	16	16	16	16	16	16	16	16	16	16	16	16	16
N-cad	Pearson correlation	-.492	-.438	-.797**	-.239	-.471	-.458	-.263	-.723**	-.790**	-.775**	-.728**	.722**	1	.815**	-.249
	Significant（Bilateral）	.053	.090	.000	.373	.065	.075	.326	.002	.000	.000	.001	.002		.000	.352
	N	16	16	16	16	16	16	16	16	16	16	16	16	16	16	16
ZO-1	Pearson correlation	-.771**	-.438	-.712**	-.568*	-.533*	-.565*	-.233	-.820**	-.814**	-.806**	-.741**	.610*	.815**	1	-.198
	Significant（Bilateral）	.000	.089	.002	.022	.033	.023	.384	.000	.000	.000	.001	.012	.000		.462
	N	16	16	16	16	16	16	16	16	16	16	16	16	16	16	16
Claudin	Pearson correlation	.139	.344	-.143	.274	.095	-.099	.015	.348	.177	.260	-.011	-.134	-.249	-.198	1
	Significant（Bilateral）	.608	.192	.598	.304	.727	.716	.955	.187	.512	.331	.969	.621	.352	.462	
	N	16	16	16	16	16	16	16	16	16	16	16	16	16	16	16

Figure S1. Original western blot gels used in the study.

Occludin (FIg.2A)	β -actin (FIg.2A)
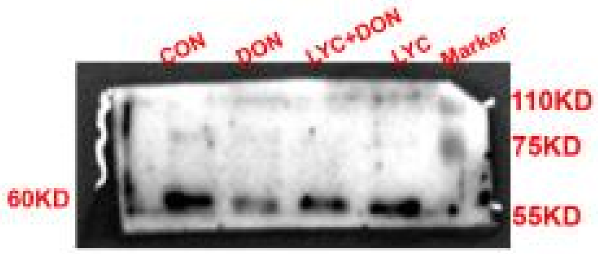	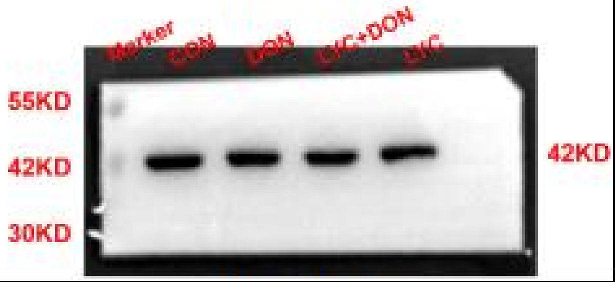
**ZO-1 (Fig.2B)**	**β -actin (FIg.2B)**
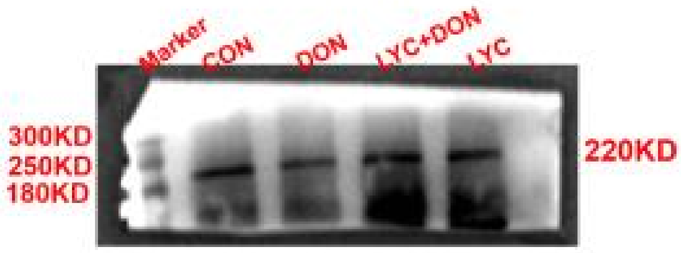	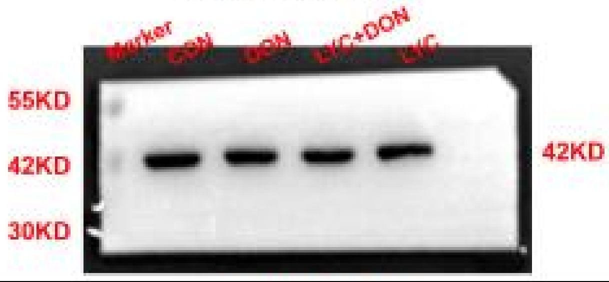
**N-cad (Fig.2C)**	**β -actin (FIg.2C)**
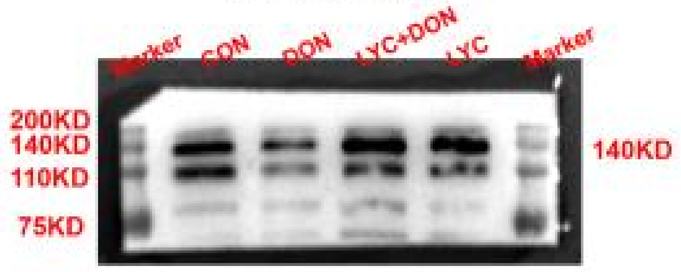	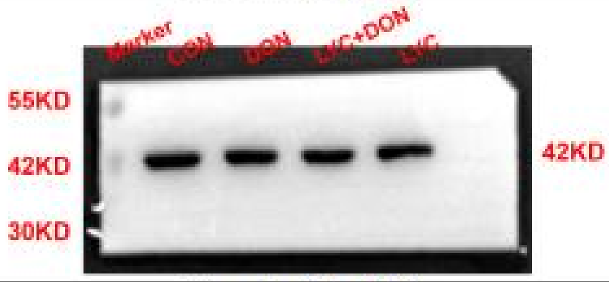
**Claudin (Fig.2D)**	**β -actin (FIg.2B)**
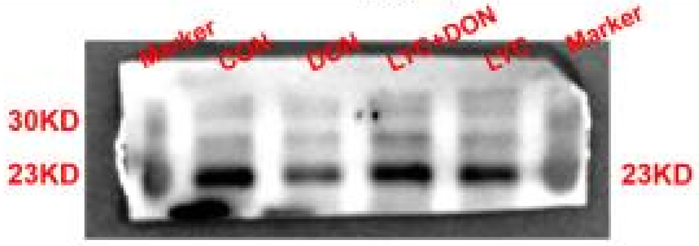	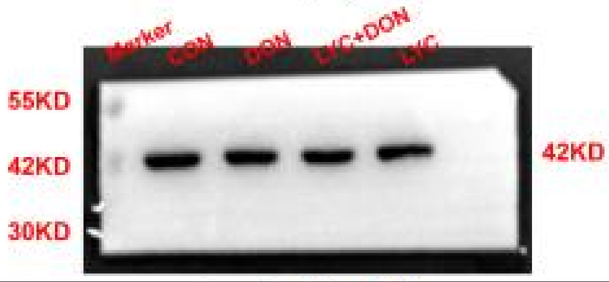
**Dnm1l (Fig.5C)**	**β -actin (Fig.5C)**
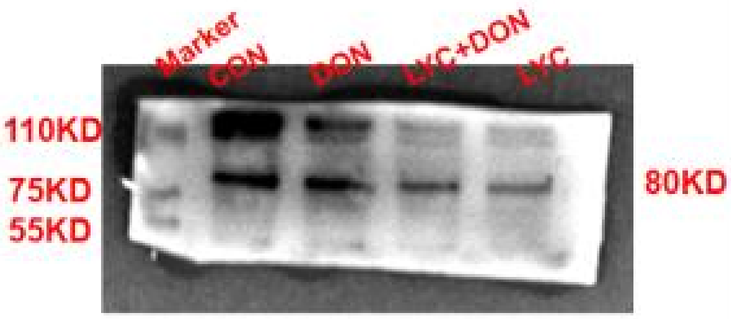	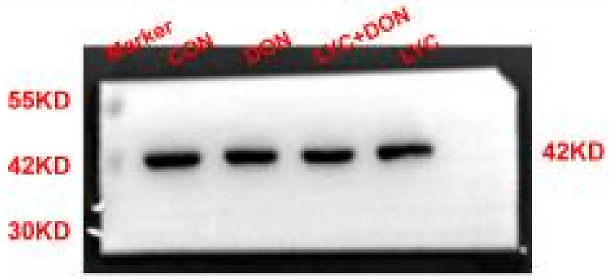

Parkin (Fig.6A)	β -actin (Fig.6A)
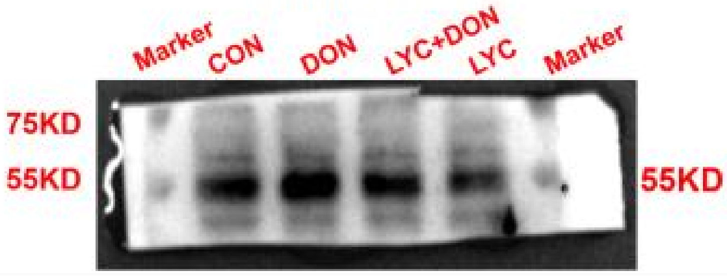	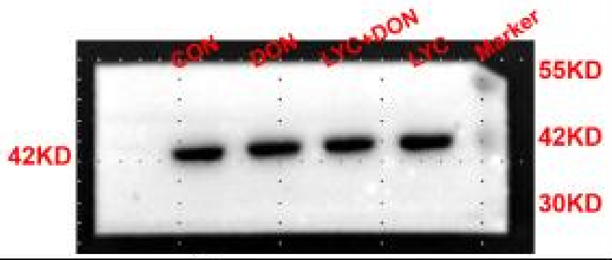
**PINK1 (Fig.6B)**	**β -actin (Fig.6B)**
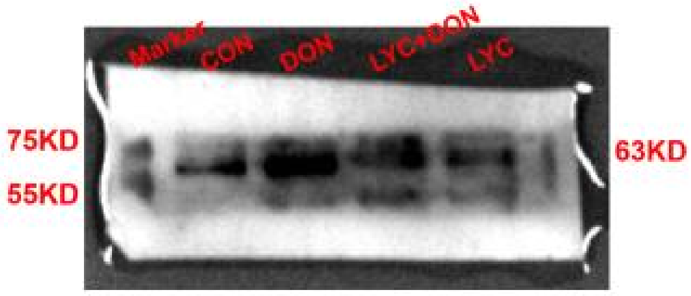	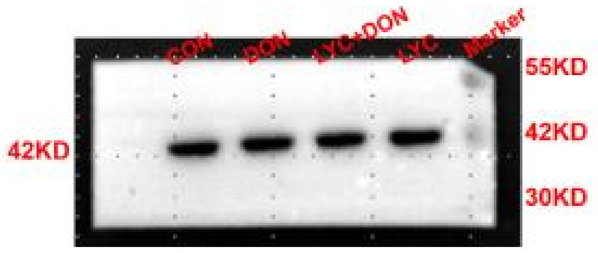
